# Structure-Based Virtual Screening Identifies Multiple Stable Binding Sites at the RecA Domains of SARS-CoV-2 Helicase Enzyme

**DOI:** 10.3390/molecules26051446

**Published:** 2021-03-07

**Authors:** Sajjad Ahmad, Yasir Waheed, Saba Ismail, Saadia Bhatti, Sumra Wajid Abbasi, Khalid Muhammad

**Affiliations:** 1 Foundation University Medical College, Foundation University Islamabad, DHA-I, Islamabad 44000, Pakistan; sahmad@bs.qau.edu.pk (S.A.); sabaismail7@gmail.com (S.I.); 2 Department of Health and Biological Sciences, Abasyn University, Peshawar 25000, Pakistan; 3 Department of Biochemistry, Faculty of Biological Sciences, Quaid-i-Azam University, Islamabad 44000, Pakistan; sfbhatti@bs.qau.edu.pk; 4 NUMS Department of Biological Sciences, National University of Medical Sciences, Abid Majeed Rd, The Mall, Rawalpindi 46000, Pakistan; sumra.abbasi@numspak.edu.pk; 5 Department of Biology, College of Science, United Arab Emirates University, Al Ain 15551, United Arab Emirates

**Keywords:** SARS-CoV-2 helicase, COVID-19, molecular dynamic simulation, phytochemicals

## Abstract

With the emergence and global spread of the COVID-19 pandemic, the scientific community worldwide has focused on search for new therapeutic strategies against this disease. One such critical approach is targeting proteins such as helicases that regulate most of the SARS-CoV-2 RNA metabolism. The purpose of the current study was to predict a library of phytochemicals derived from diverse plant families with high binding affinity to SARS-CoV-2 helicase (Nsp13) enzyme. High throughput virtual screening of the Medicinal Plant Database for Drug Design (MPD3) database was performed on SARS-CoV-2 helicase using AutoDock Vina. Nilotinib, with a docking value of −9.6 kcal/mol, was chosen as a reference molecule. A compound (PubChem CID: 110143421, ZINC database ID: ZINC257223845, eMolecules: 43290531) was screened as the best binder (binding energy of −10.2 kcal/mol on average) to the enzyme by using repeated docking runs in the screening process. On inspection, the compound was disclosed to show different binding sites of the triangular pockets collectively formed by Rec1A, Rec2A, and 1B domains and a stalk domain at the base. The molecule is often bound to the ATP binding site (referred to as binding site 2) of the helicase enzyme. The compound was further discovered to fulfill drug-likeness and lead-likeness criteria, have good physicochemical and pharmacokinetics properties, and to be non-toxic. Molecular dynamic simulation analysis of the control/lead compound complexes demonstrated the formation of stable complexes with good intermolecular binding affinity. Lastly, affirmation of the docking simulation studies was accomplished by estimating the binding free energy by MMPB/GBSA technique. Taken together, these findings present further in silco investigation of plant-derived lead compounds to effectively address COVID-19.

## 1. Introduction

The coronavirus disease 2019 (COVID-19) pandemic has drastically affected almost 218 countries while imposing a severe health and economic burden [1]. A novel coronavirus (nCoV, SARS-CoV-2) is reported to be the causative agent of this infectious disease with the mode of transmission of COVID-19 being found to be through nasopharyngeal discharge from the nose, including droplets of saliva expelled during sneezing or coughing by an infected person [2,3]. Despite the non-specificity of the symptoms and asymptomatic condition of the disease, a range of prevailing acute symptoms such as dry cough, loss of smell or taste, fever, fatigue, diarrhea, sore throat and body aches are the hallmark features of this viral disease [4,5]. In patients with chronic conditions, severe acute respiratory syndrome pneumonia followed by multi-organ infection leading to death has been reported [6]. Many broad-spectrum antiviral drugs and new vaccines are still pending approval by the WHO panel for the subsequent symptomatic management and prevention of COVID-19 [7,8,9,10,11].

Startlingly, coronaviruses (CoVs) have long been considered a serious threat to both mammals and birds, causing severe enteric and respiratory infections before becoming a global health burden in 2002 [12,13]. They are, therefore, categorized into four different genera viz. alpha-, beta-, gamma-, and delta-COVs [14,15]. Due to their genomic susceptibility towards high mutational and recombination rates, new strains, each having unique virulence, continue to emerge [12]. To date, around seven different strains of human CoVs are reported, namely, 229E, NL63, OC43, HKU1, Middle East respiratory (MERS)-CoV, severe acute respiratory syndrome (SARS)-CoV, including the currently evolving 2019-novel coronavirus(nCoV) [13]. These viruses share common primary sites of infection, i.e., the upper and lower respiratory tract and cause symptoms ranging from mild colds to severe respiratory conditions such as pneumonia, bronchiolitis, rhinitis, pharyngitis, and sinusitis [16].

With the epidemics of high morbidity caused by SARS-CoV in 2003 and MERS-CoVs in 2012, together with their adaptability towards drastically changing environment, CoVs are now categorized as “emerging viruses”. They are enveloped, positive-sense, single-stranded RNA (+ssRNA) having a genomic size ranging from 26.2 to 31.7 kb [12]. Structurally, they have a “crown-like” appearance as revealed by electron micrographs due to club-shaped peplomers projecting outwards like spikes [17]. Belonging to a β genus, SARS-CoV-2 consists of both nonstructural proteins (NSPs) as well as structural proteins, namely; Membrane (M), Spike (S), Envelope (E) and Nucleocapsid (N) proteins [8]. Interestingly, one of the major targets of neutralizing antibodies is the spike surface glycoprotein, which is primarily involved in host attachment and in the subsequent viral-host cell membrane fusion to initiate the viral infection cycle [18].

The helicase enzyme, which is a motor protein, is an example of one such NSP that drives the unwinding of double-stranded nucleic acids along the 5′-3′ direction during biological processes, including recombination replication and repair. This unwinding results in converting them into two single-stranded RNAs. Helicases are known to utilize the energy released during nucleotide hydrolysis to facilitate these activities [19,20]. Recent literature surveys have reported the additional biological role of helicases, including transcription, mRNA splicing, mRNA export, RNA stability, translation, mitochondrial gene expression, and nucleic acid packaging into virions [21,22]. A recent study has experimentally confirmed the strategic targeting of SARS-CoV-2 helicases using reported antiviral drugs as evident from the in vivo findings on the inhibition of herpes simplex virus (HSV)-encoded helicases in animal models [23]. Like both SARS-CoV and MERS helicases, SARS-CoV-2 helicase is a triangular pyramid-shaped enzyme, 596 amino acids long with five domains [20,21]. These domains consist of two RecA-like domains (1A and 2A) towards the core of C-terminal Helicase, the N-terminal zinc binding domain (ZBD), and the β-barrel domain (1B), with the stalk domain connecting 1B and ZBD [24].

The NTP hydrolytic activity is attributed to six key residues (Lys288, Ser289, Asp374, Glu375, Gln404 and Arg567) found within the cleft between the 1A and 2A domains at the base. These residues are located at the active site of SARs-CoV-2 helicase enzyme [25,26]. This implies that NTPase inhibition via disruption of ATP binding by small molecules could be a promising strategy for novel helicase inhibitors [27]. Fpocket, a computer-aided algorithm, was used to predict and shortlist pocket 26 on the allosteric site, a potent inhibitory target site for a reference hydrocarbon compound called triphenylmethane. The residues, namely; Leu132, Leu235, Glu136, Phe133, Pro234, Arg22, and Arg129 are integral part of Pocket 26, with pocket 25 being another potential target of a helicase inhibitor, Darunavir with antiviral activity. Likewise, a number of other plant derived natural compounds were identified as helicase inhibitors in vitro, particularly flavonoids such as xanthones, rutin, triptexanthoside D, phyllaemblinol and quercetagetin [10]. Other effective inhibitors of SARS-CoV helicases including myricetin, scutellerein, eubananin, bananin, vanillinbananin, and iodobananin are also reported. These compounds work by blocking the ATPase activity rather than through the unwinding activity [28,29]. Besides natural products with inhibitory activity against SARS helicase enzyme, synthetic chemical compounds are also reported and these include; 7-ethyl-8-mercapto-3-methyl-3,7-dihydro-1*H*-purine-2,6-dione, SSYA10-001, a 1,2,4 triazole, and (*E*)-3-(furan-2-yl)-*N*-(4-sulfamoylphenyl)acrylamide [30,31,32].

Many other FDA-approved antiviral drugs have also shown promising inhibitory activity against helicases. The drugs that are so far predicted to be repurposed for the treatment of COVID-19 include anticoagulants (dabigatran), antifungals (itraconazole), anti-bacterials (lymecycline, cefsulodine and rolitetracycline), diuretics (canrenoic) and anti-HIV-1 drugs (saquinavir) [8,10,33]. Computer-aided drug discovery, design and development of small molecules against viral protein targets, therefore, offers a fast-paced, cost-effective approach [34]. Among the viral protein targets required for the design of small-molecule agents with inhibitory activity, SARS-CoV-2 helicase (nsp13) is of particular interest due to its highly conserved genomic sequences across coronaviruses, besides its unique function, and characteristic active site [35]. Given all this, using different applications of computational drug design herein we virtually screened Medicinal Plant Database for Drug Designing (MPD3) database [36] against SARS-CoV-2 helicase to identify new phytochemicals with improved binding, pharmacokinetics, non-toxicity and easily available for experimentalists for in vitro and in vivo testing.

## 2. Materials and Methods 

**Figure 1:**
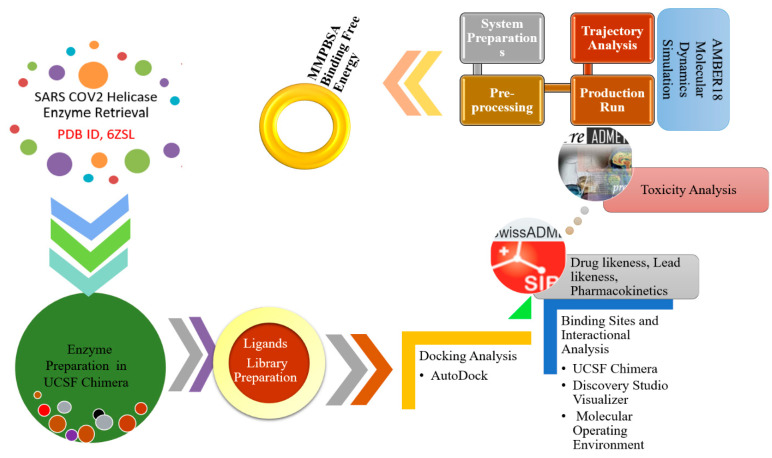
Flowchart of the methodology employed in the present study.

A summary of the methodology flow used in this study for the identification of hit and stable molecules against SARS-CoV-2 helicase is presented in Figure 1. 

### 2.1. Preparation of the SARS-CoV-2 Helicase Structure

The study commenced with the retrieval of SARS-CoV-2 helicase enzyme crystal structure (PDB ID, 6ZSL) available at a good resolution of 1.94 Å. Immediately, the structure was treated in UCSF Chimera, alpha version 1.15 [37] minimization phase where its geometry was optimized, loops and side chains were fixed and hydrogen atoms were added. All co-crystalized ligands were deleted and the structure energy was minimized via two-step process to remove high energies. It was noticed that minimization of 500 steps of steepest descent steps and 500 conjugate gradient steps at step size of 0.02 Å are enough to get high stereo-chemical quality of the enzyme close to the native structure. 

### 2.2. Phytochemicals Library Preparation

For virtual screening, MPD3 (https://www.bioinformation.info/index.html) accessed on 10 September 2020 was used [36]. This database is freely available, downloadable and contains information pertaining to phytochemicals, their structures and activities and test targets. Currently, the MPD3 contains 12,281 phytochemicals which are grouped into several categories, i.e., aromatics, alkaloids, steroids, saponins, flavonoids, etc. The complete library was downloaded and imported to the PyRx virtual screening package 0.8 [38] where all compounds were energy minimized and converted to pdbqt format. Nilotinib, which is a tyrosine kinase inhibitor, was used as a control molecule. This molecule has been demonstrated to inhibit SARS-CoV-2 in vitro [39] and interacts with SARS-CoV-2 helicase enzyme [19].

### 2.3. Binding Conformational Analysis

AutoDock 4.2 [40] was utilized to dock the control inhibitor (nilotinib) as well as library of phytochemicals from Nsp13 helicase enzyme protein towards the whole protein surface. The grid box was centered at x: −13.62, y: 26.04 and z: −70.09 coordinates, with the dimensions of the grid points set to 69.75 × 86.68 × 68.21. The grid spacing for this enzyme was adjusted to 0.375 Å. The Lamarckian genetic algorithm (LGA) was then used for the molecular docking with its specified parameters set to default as follows; initial population size; 150 individuals, maximum number of generations: 27,000, maximum number of energy evaluations: 2,500,000, with 0.02 gene mutation rate, cross over rate of 0.8 with number of runs set to 100 GA. The root mean square deviation (RMSD) [41] having a threshold value of 2.0 was used for binding conformational studies, with the lowest inhibition constant values and the lowest binding energy considered as the most favorable binding conformation. UCSF Chimera, alpha version 1.15 [37], Discovery Studio Visualizer [42] and Molecular Operating Environment (MOE) [43] programs were used to analyze the conformational binding and molecular basis of interactions between the enzyme and ligands. Drug-likeness, pharmacokinetics, and toxicity profiles of hits were then unraveled through SwissADME [44] and PreADMET [45]. 

### 2.4. Molecular Dynamics (MD) Simulation

To understand and assess the chemical, biological, physical, as well as structural stability, it was crucial to analyze the conformational behavior of the screened ligand and its complexes with the SARS-CoV-2 helicase enzyme [46]. The AMBER18 program [47] with the general AMBER force field [48] for ligands preparation and the ff14SB force field [49] for enzyme preparation were used during the molecular simulation to evaluate the dynamic and structural profiles of ligands docked into the binding sites of the target protein of interest. After initial preparation, each system was subjected to 500 steps of steepest descent and conjugate gradient minimization steps. The immersion of top-selected docking complexes was performed in TIP3P water box (the spacing between the edge box and complex was adjusted at 12.0 Å. Counterion treatment was done for system neutralization. The NVT ensemble was run for 20 ps to heat the system to a target temperature set to 310 K. Consequently, NPT ensemble was applied to the system for approximately 40 ns to equilibrate the system, followed by 50 ns of production simulation. The pressure was maintained at an average of 1 atm using isotropic position scaling. Temperature controlled was accomplished via Langevin dynamics allowing the collision frequency of 1 ps^−1^ [50]. For non-bonded interactions, a cutoff of 8 Å was used, while for long range electrostatic interaction, Particle Mesh Ewald (PME) method was employed [51]. The hydrogen bonds were constrained by SHAKE method [52]. Lastly, the generated MD trajectories were analyzed through CPPTRAJ [53] and Visual Molecular Dynamics (VMD) v.1.9.3 [54].

### 2.5. Free Binding Energy Calculations via MMPB/GBSA 

The binding free energy calculations were performed using a force field-based approach through a MMPB/GBSA method [55]. This is used to calculate the difference in binding free energy resulting from the interactions between the ligands (small molecules), protein (macromolecular target) and the solution complex free energies [56]. These intermolecular activities between the small molecules and their ability to bind to the target protein is mathematically equated as follows:

L + P → LP

Where the symbols ‘L’ and ‘P’ represent the ligand and target protein and the complex is represented by ‘LP’. In principal, this in silico approach provides useful details on the assessment of the free energy of this reaction as represented by **ΔG_bind_**. Thereby, predicting the binding affinity of any drug without the need to experimentally synthesize it first. The following equation is computed for the calculation of free binding energy:

ΔG_bind_ = G_LP_ − (G_P_ + G_L_)

The mathematical relationship between the free energy associated with the ligand, proteins and their complexes, with their decomposition state into the gaseous phase, MM energy, including the nonpolar and polar solvent and entropy are represented by the following formula:

ΔG = ΔE_MM_ + ΔG_solv_ − T·ΔS = ΔE_BAT_ + ΔE vdW + ΔE coul + ΔG solv,p + ΔG solv,np − T·ΔS

The sum of bond, torsion and angle terms in the force field are collectively denoted by E_BAT_, and E_MM_, whereas E_vdW_, and E_coul_ represent the van der Waals term and Coulombic term, respectively. The generalized-Born (GB) approximation is used for the estimation of the solvation free energy, where G_solv,np_ denotes the nonpolar solvation free energy, which is a linear function of a computational interface; solvent-accessible surface area (SASA). Then, the VSGB 2.0 solvation model/ MMPB/GBSA energy model was used to calculate the binding energies of ligand-protein complexes, neglecting the entropy term [57]. The net MMPB/GBSA energy associated with each screened compound which is estimated through the 100-trajectory frames collected per simulation run. 

## 3. Results and Discussions

### 3.1. Virtual Screening of MPD3 Database

**Figure 2:**
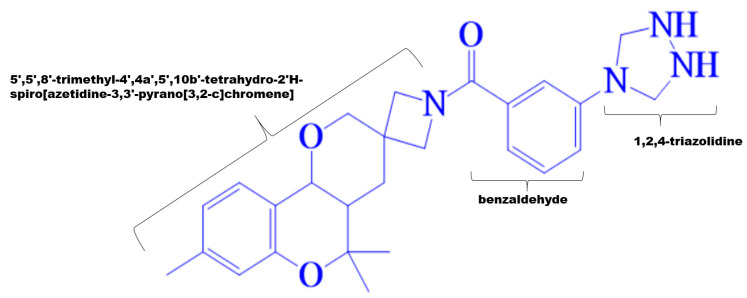
Structural dissection of the hit molecule virtually screened against SARS-CoV-2 helicase enzyme.

The proposed study involved the virtual screening of MPD3 phytochemical library against SARS-CoV-2 helicase enzyme through a combination of docking, MD simulations and MMPB/GBSA methods. Approximately 1131 compounds were shortlisted based on their excellent molecular docking (binding affinity < −7 kcal/mol) with the target protein structure. Subsequently, the PubChem CID: 110143421, ZINC database ID: ZINC257223845, eMolecules: 43290531 or (3-(1,2,4-triazolidin-4-yl)phenyl) (5′,5′,8′-trimethyl-4′,4a′,5′,10b′-tetrahydro-2′*H*-spiro[azetidine-3,3′-pyrano[3,2-c]chromen]-1-yl)methanone small molecule inhibitor (binding energy; −10.2 kcal/mol) was ranked as best binder to the ATP binding site of SARS-CoV-2 helicase enzyme. In compare, the control, nilotinib has a scoring value of −9.6 kcal/mol during the docking procedure. The 2D structure of the hit molecule is shown in Figure 2.

### 3.2. Comparative Binding Sites and Conformational Analysis

**Figure 3:**
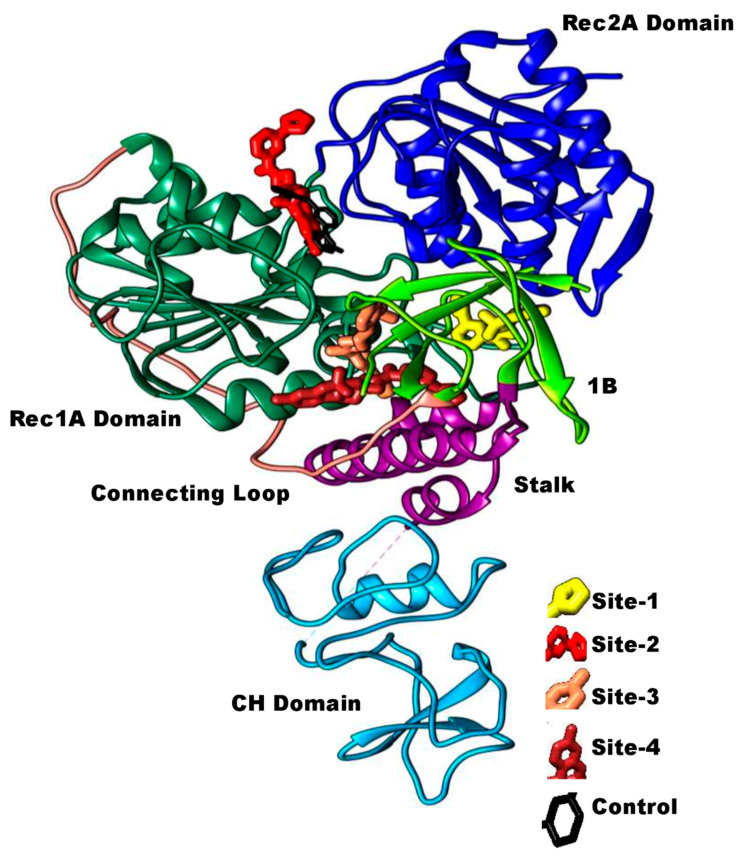
The different binding sites and conformation of the top ranked compound filtered in this study. Control binding site is also provided.

The top ranked compounds and controls were examined for their natural tendency of binding to the SARS-CoV-2 helicase enzyme. In all docking iterations, the top ranked compound demonstrated to show binding at different sites of triangular based collectively formed by RecA domains (1A and 2A) and 1B domain (Figure 3). The control (black stick), on the other side, prefers docking only at the ATP binding region of the triangular base. Among the docked sites for the virtually screened PubChem CID, 110143421 compound, the hotspot is the ATP binding site (binding site 2) like that of control. The 4-phenyl-1,2,4-triazolidine group of the compound is posed to the cavity between Rec1A and Rec2A domains where its 1,2,4-triazolidine titled more towards Rec2A domain. The opposite 5′,5′,8′-trimethyl-4′,4a′,5′,10b′-tetrahydro-2′H-spiro[azetidine-3,3′-pyrano[3,2-c]chromene]-1-carbaldehyde chemical structure of the compound accommodates itself at the ATP binding site of Rec1A domain. The three other binding sites of the compound are at the interface cavity between Rec1A and 1B domains with stalk at the base (binding site 3 and 4), and Rec1A loop (between helix 14 and helix 15) at the base of Rec2A and adjacent to 1B domain (binding site 1). At the Rec1A and 1B domains interface, the compound was observed aligned either vertically along the pocket or horizontally alongside the base stalk.

### 3.3. Comparative Chemical Interactions Analysis

**Figure 4:**
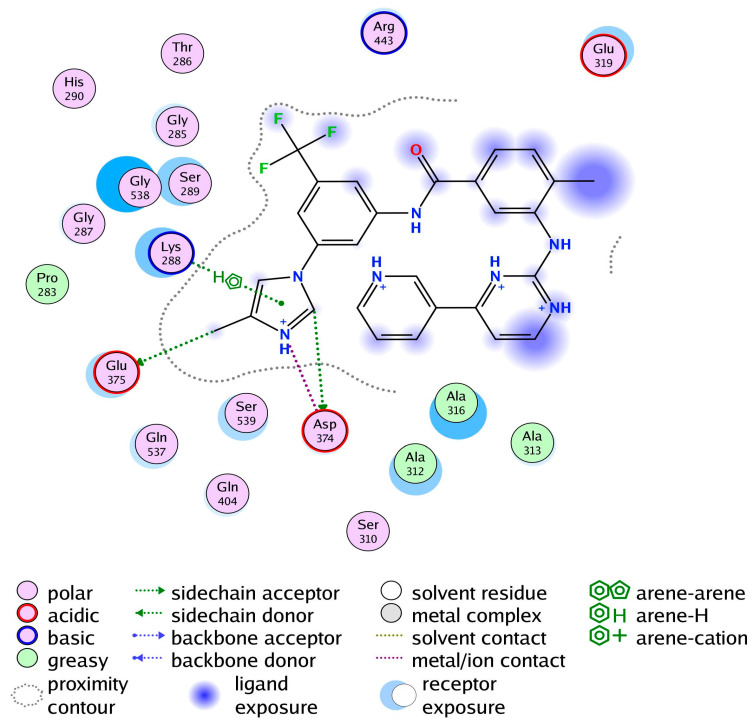
Binding interaction network of the control with SARS-CoV-3 helicase enzyme.

**Figure 5:**
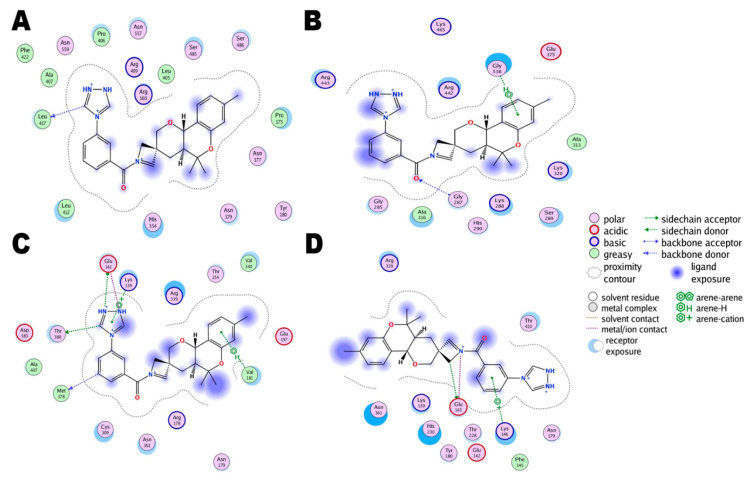
Binding interactions of the top ranked compound docked at different binding sites of the SARS-CoV-2 helicase enzyme. (**A**). Site 1, (**B**). Site 2, (**C**)**.** Site 3, and (**D**). Site 4.

Next, molecular-level interactions involved in binding the compound/control at different sites of the SARS-CoV-2 helicase enzyme were investigated to decipher the key chemical forces crucial for intermolecular binding and stability of complexes. The control Nilotinib at the ATP binding site is reported to form strong hydrogen bonds, in particular with the enzyme H9 helix residues (Gly287, Lys288, and Ser289) at Rec1A domain via its (trifluoromethyl)benzene. The rest of the compound structure stabilization is provided by medium and long range van der Waals and other hydrophobic interactions (Figure 4). The lowest binding energy conformation of the compound at site 1 is anchored at the H14-H15 helix Rec1A domain loop, with further chemical stabilization by dual hydrogen bonds with Asn557 of Rec2A via 1,2,4-triazolidine ring (Figure 5A). The predominant ATP binding site (binding stie 2) of the hit compound involved mainly van der Waals bonding and alky interactions at the binding site of Rec1A and Rec2A domains throughout the length of the compound (Figure 5B). At binding site 3, the conformation of the compound produces two hydrogen bonds through its 1,2,4-triazolidine ring with Glu142 stalk H5 helix, the acetophenone is attached to Rec1A domain through a single hydrogen bond with Asn361, and 3,3,5,5,8-pentamethyl-2,3,4,4a,5,10b-hexahydropyrano[3,2-c]chromene also formed a hydrogen bond with Arg339 Rec1A H11-H12 loop. The remaining compound structure established multiple van der Waals, sigma and alkyl interactions with Rec1A, stalk and 1B domains (Figure 5C). In the least determined binding conformation (binding site 4), 1,2,4-triazolidine ring again engaged Glu142 of the stalk H5 helix in hydrogen bonding while the remaining chemical moieties are hydropophically attached with Rec1A, stalk and 1B domains (Figure 5D).

### 3.4. SwissADME Analysis

**Figure 6:**
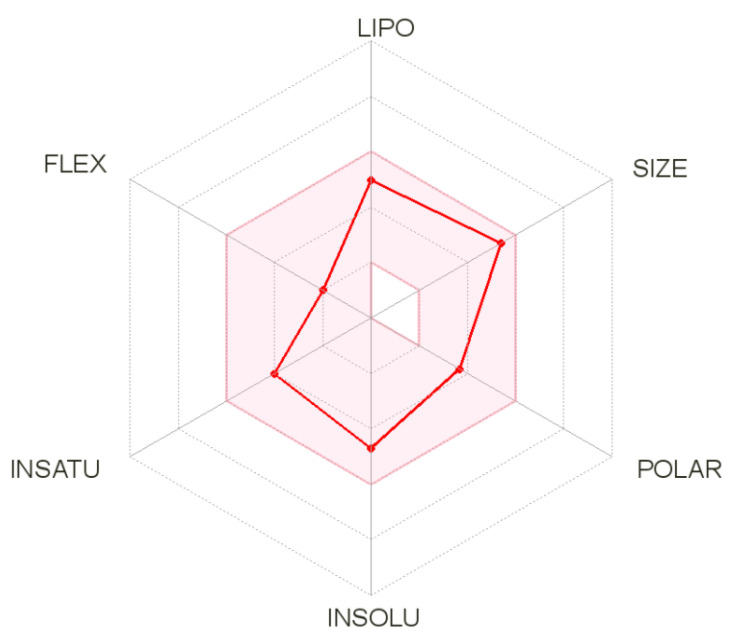
Oral bioavailability radar of the compound (shown by red line). The pink color zone represents a suitable physicochemical space for oral bioavailable drugs. INSATU (instauration), POLAR (polarity), INSOLU (insolubility), LIPO (lipophility), FLEX (flexibility), and SIZE (molecular weight).

SwissADME is an online server used to compute different physicochemical descriptors along with predictions of drug-like nature, ADME parameters, medicinal chemistry friendliness and pharmacokinetic properties to assist drug discovery. Detail results of each term for the hit molecule described above are listed in Table 1. The oral bioavailability radar of the compound is presented in Figure 6. Physicochemically, the compound properties are within the range of drug-likeness and do not violate any of the Lipinski rule parameters. Topological polar surface area (TPSA), which is the surface sum of all polar atoms, is commonly used metrics to optimize drug capacity to penetrate the blood barrier [58]. Moreover, the compound has good lipophilicity thus maximizing its transportation and reaching to the target site [59]. Additionally, the compound has good gastrointestinal absorption and does not inhibit the majority of the cytochrome P450 isoforms that are significant in drug elimination through the process of metabolic biotransformation. The compound was also demonstrated to fulfill all requirements of the prominent Lipinski [60], Veber [58], Egan [61] and Muegge [62] druggability rules. The bioavailability score of the compound is 0.55. This predicts the compound probability to be at least 10% bioavailable. From a synthetic chemistry perspective, the compound synthesis is easy. The molecule also predicted not to contain Pan-assay interference compounds (PAINS) alerts and will not interact non-specifically with multiple biological targets but rather react with one specific desired target [63]. More importantly, the compound is non-toxic.

### 3.5. MD Simulation of the Docked Models for Structural Stability Analysis

**Figure 7:**
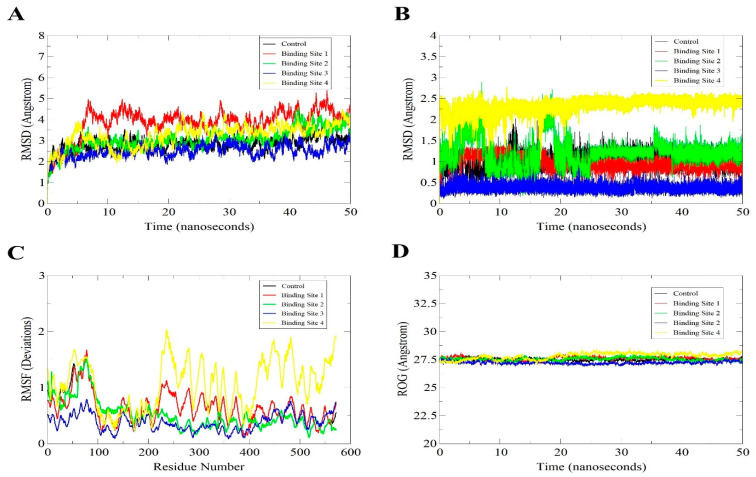
MD simulation-based analysis of structural and stability complexes. (**A**). Enzyme RMSD analysis, (**B**). Ligand RMSD analysis, (**C**), Enzyme RMSF analysis and (**D**), Enzyme ROG analysis.

With the docked model having the highest stability profile, MD simulation was conducted with a run-time of 50 ns. Then, using root-mean-square deviation (RMSD) of the SARS-CoV-2 helicase and control/compound as shown in Figure 7A,B, the structural stability analysis were performed on the docked models. The mean RMSDs and standard deviations of the enzyme structure in all complexes are as; control (2.86 Å ± 0.33), binding site 1 (3.84 Å ± 0.66), binding site 2 (3.07 Å ± 0.53), binding site 3 (2.52 Å ± 0.31) and binding site 4 (3.26 Å ± 0.52). Furthermore, ligands mean RMSDs and standard deviations values in these complexes are; control (1.04 Å ± 0.19), binding site 1 (0.99 Å ± 0.15), binding site 2 (1.19 Å ± 0.33), binding site 3 (0.37 Å ± 0.08) and binding site 4 (2.34 Å ± 0.17). The conformations derived from the VMD analysis revealed the inhibitors were constantly attached to the binding sites of target proteins in the complex. Furthermore, any changes of residues as well as the similar patterns with fluctuations within complexes were identified using root mean square fluctuations (RMSF) (Figure 7C). RMSD analysis indicated that the binding site 2 (ATP) binding site is more comparable to the control and has the same stability pattern. In contrast, the complex of the enzyme and compound at binding site 3 demonstrated high residual flexibility. The compound binding site at 4 was observed to induce more residual flexibility but still highly within the acceptable range. The highly fluctuating regions revealed the residues Thr228–Val570 present towards the active site with highly flexible loops, as shown in (Figure 4C). Hence, the stability of the docked models were confirmed by both the RMSD and RMSF analyses. Similarly, the helicase enzyme in all complexes is highly compact and can be concluded to enjoy structural stability in the enzyme presence (Figure 7D). The mean ROG values for the complexes are; control (27.50 Å ± 0.11), binding site 1 (27.57 Å ± 0.15), binding site 2 (27.52 Å ± 0.14), binding site 3 (27.22 Å ± 0.11) and binding site 4 (27.82 Å ± 0.27).

### 3.6. Protein-Inhibitor Stability Involving Hydrogen Bond Interactions

**Figure 8:**
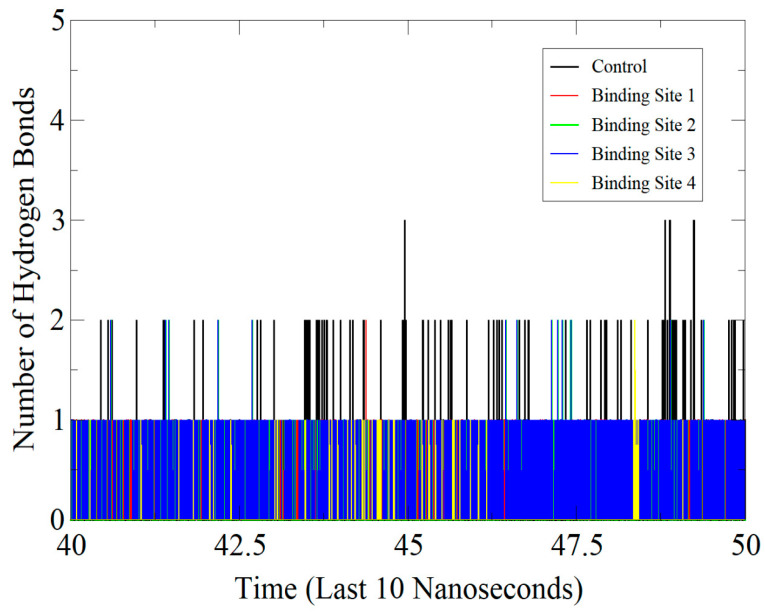
Hydrogen bond analysis for control/hit complexes in the last 10 ns of MD simulation trajectories.

The MD simulations were also performed to study the effect of hydrogen bond interaction by measuring the distances between the hydrogen bond (usually heavy atoms) donors and acceptors [64]. This further provided the number and specific binding patterns between the control/compound and enzyme as shown by the active sites given in Figure 8. The control was inferred to be engaged in a network of strong hydrogen bonds (maximum 3) with close distances from the ATP site throughout the simulation time, in particular getting stronger towards the end. Likewise, the predominant ATP binding site of the compound (site 2) seems to follow the same binding pattern of control and demonstrated favor formation of close distance hydrogen bonding. At the binding site 3, it was observed during the simulation procedure that the number of hydrogen bonding and distances were fluctuating, such alterations, however, did not influence the interaction capacity of the binding compound. The binding site 4 of the compound was found the most unstable in terms of hydrogen bonds and the binding patterns seemed highly fluctuating.

### 3.7. Determining Binding Free Energies

The MD trajectories were utilized to estimate the total and residual binding free energies associated with the interactions between the control/compound and the enzyme. During the trajectory analysis, the main interacting residues involved in inhibitor-bound conformation were determined. From the 100 frames of MD trajectories, all complexes including the control had similar and stable net binding energy values indicating stable binding of the control as well as that of the compound at different binding sites of the SARS-CoV-2 helicase triangular base. Comparatively, in the case of MMGBSA, the control compound complex had better net binding energy value. On the contrary, the filtered high affinity binding at site 1 had the stronger binding, followed by site 4th, 3rd and predominant ATP binding site. In all these complexes, higher contribution was found from the gas phase with significant stability provided by van der Waals energy and sufficient by electrostatic energy. For the stronger binding, the nonpolar energy was also demonstrated to play some role in the complex binding.

Table 2 shows the MMGBSA statistical analysis achieved through all MD trajectory frames during the simulations, which maintained the stability of the interactions between the control/compound and the target protein. These stable interactions are −57.72 ± 2.20 kcal/mol for control, −51.75 ± 2.84 kcal/mol for site 1, −36.17 ± 4.07 kcal/mol for site 2, −44.84 ± 2.87 kcal/mol for site 3, and −47.54 ± 3.43 kcal/mol for site 4, all indicating higher binding interactions.

The given Table 1 also showed that the average MMPBSA binding energy value (−41.52 ± 5.29 kcal/mol) for control, was found to be lower at site 1 of the compound (−45.78 ± 3.54 kcal/mol). While, the binding free energy for site 2, site 3 and site 4 are −32.18 ± 4.52 kcal/mol, −34.16 ± 4.40 kcal/mol, −39.16 ± 4.04 kcal/mol, respectively. A similar trend of higher van der Waals and less electrostatic contribution as reported in the MMGBSA was also seen in the MMPBSA. Again, the net gas phase energy is dominated by both the control and compound binding to the SARS-CoV-2 helicase enzyme in contrast to the non-favorable role of the solvation energy.

### 3.8. Residue Wise Energy Contribution

To gain further insight into the role of individual residues in the binding pockets of the compound/control, the MMGBSA binding free energy was decomposed per residue. It was observed that the majority of the interacting residues of the control and the screened compound are located within the hydrophobic pocket towards the binding site and have shown moderate interactions with the ligand molecule and hence moderate binding affinities as predicted by their docking analysis. In case of control that binds to the ATP binding site, the strongest residues average free energy were those of Gly518 (−3.41 kcal/mol), Glu355 (−3.15 kcal/mol), Ala293 (−2.94 kcal/mol), Gln384 (−1.98 kcal/mol), Lys268 (−1.90 kcal/mol), Ser519 (−1.45 kcal/mol), Pro264 (−1.43 kcal/mol), Leu297 (−1.13 kcal/mol), Ala292 (−1.04 kcal/mol), and Ser290 (−1.03 kcal/mol). All these mentioned residues are either within the close proximity of the binding site of the control drug or lie within the binding pocket. The control drug is reported to contribute heavily towards the complex energy and it is −32.39 kcal/mol. The most prevalent binding site of the filtered high affinity binder which binds to the same site with that of the control drug had a net binding energy of is −21.63 kcal/mol and stabilized by residues Arg422 (−3.2 kcal/mol), Glu241 (−2.61 kcal/mol), Hie270 (−2.40 kcal), and Gly267 (−1.93 kcal/mol). Contributing residues of compound binding site 1 were found to be Asn537 (−2.70 kcal/mol), Arg540 (−2.65 kcal/mol), Hie534 (−2.62 kcal/mol), Pro386 (−2.29 kcal/mol), Leu392 (−1.98 kcal/mol), Leu397 (−1.88 kcal/mol), Thr396 (−1.47 kcal/mol), Thr393 (−1.14 kcal/mol), Arg389 (−1.02 kcal/mol) while the compound itself had binding energy of −27.76 kcal/mol. For the binding site 3, the following residues: Arg389 (−2.10 kcal/mol), Thr390 (−2.09 kcal/mol), Leu130 (−1.96 kcal/mol), Glu134 (−1.82 kcal/mol), Thr360 (−1.78 kcal/mol), Ala387 (−1.65 kcal/mol), Met358 (−1.33 kcal/mol), Lys131 (−1.30 kcal/mol), Cys289 (−1.28 kcal/mol), Leu391 (−1.09 kcal/mol) were vital in stabilizing the compound binding. The net binding energy of the compound at this site is −23.85 kcal/mol. Furthermore, the binding site 4 residues Tyr172 (−3.35 kcal/mol), Pro388 (−2.16 kcal/mol), Ala387 (−1.97 kcal/mol), Glu134 (−1.96 kcal/mol), Thr390 (−1.65 kcal/mol), Met358 (−1.44 kcal/mol), Asn171 (−1.39 kcal/mol), Arg389 (−1.33 kcal/mol), Lys138 (−1.31 kcal/mol), and Leu391 (−1.02 kcal/mol) played a vital role in inducing the binding affinity of the compound via hydrophobic and electrostatic interactions. At this binding site, the compound achieved a binding energy of −25.79 kcal/mol.

## 4. Conclusions

Due to the alarming increase in transmissibility and infectivity rate of SARS-CoV-2, the development of new antiviral therapies remains a serious and demanding challenge. The SARS-CoV-2 helicase is an integral part of the virus replication machinery, does not show any sequence homology and coverage to the human proteome [65], and its crystal structure has been determined previously through X-ray crystallography. All this make SARS-CoV-2 enzyme an attractive biological target for inhibitory molecules design. Our present in silico study focused on identifying biologically-active phytochemicals that interact exclusively and with high affinity with the selected enzyme. To study the nature of these interactions as well, the insights into vital contributing residues that facilitated binding between the target protein and the control/compound, docked models were generated. The docking runs revealed that the top ranked filtered compounds and controls tend to bind to the ATP binding site of SARS-CoV-2 helicase enzyme. The binding mode of each ligand-protein docked complex was then subjected to an extensive molecular dynamic analysis. We then gathered further computational details to characterize the key residues that contribute towards binding affinity. The parameters such as the binding free energies associated with each residue towards their respective active sites were then estimated. Interestingly, it was found that the binding free energies of the intermolecular hydrogen bonding at the binding pocket showed relatively weaker contributions to the binding. On the contrary, the binding free energy derived from the hydrophobic interactions relatively showed a greater binding strength reflecting stronger interactions between the compounds bound to the helicase enzyme. Overall, during the docking process, the compounds showed a tendency of binding to three different sites of the helicase enzyme: the predominant binding site is the ATP molecule binding site (binding site 2) where both the control drug and hit compounds of this study bind. The binding site 1 is H14-H15 helix, Rec1A domain loop. On this site, the compound bounded with high affinity but were seen in fewer docking runs compared to binding site 2. The 3rd and 4th binding sites between Rec1A and Rec2A, on the other hand, are the least reported sites for compound binding. Interesting, it was inferred that the four sites were crucial in enhancing the binding of the compound to the enzyme without contributing towards the hydrogen bonding. It was further observed that the complexes are quite stable from an energy perspective, and several residues at the docked sites of the enzyme are engaging the compound strongly via van der Waals force and less by hydrogen bonding.

## Figures and Tables

**Table 1:** SwissADME and preADMET analysis of the hit molecules screened in this study.

Physicochemical Properties		Pharmacokinetics	
Formula	C26H32N4O3	GI absorption	High
Molecular weight	448.56 g/mol	BBB permeant	Yes
Num. heavy atoms	33	P-gp substrate	Yes
Num. arom. heavy atoms	12	CYP1A2 inhibitor	No
Fraction Csp3	0.5	CYP2C19 inhibitor	No
Num. rotatable bonds	3	CYP2C9 inhibitor	No
Num. H-bond acceptors	5	CYP2D6 inhibitor	Yes
Num. H-bond donors	2	CYP3A4 inhibitor	Yes
Molar Refractivity	140.83	Log *K*_p_ (skin permeation)	−6.77 cm/s
TPSA	66.07 Å²	Druglikeness	
Lipophilicity		Lipinski	Yes; 0 violation
Log *P*_o/w_ (iLOGP)	3.5	Ghose	No; 1 violation: MR > 130
Log *P*_o/w_ (XLOGP3)	3.19	Veber	Yes
Log *P*_o/w_ (WLOGP)	1.37	Egan	Yes
Log *P*_o/w_ (MLOGP)	2.92	Muegge	Yes
Log *P*_o/w_ (SILICOS-IT)	2.68	Bioavailability Score	0.55
Consensus Log *P*_o/w_	2.73	Medicinal Chemistry	
Water Solubility		PAINS	0 alert
Log *S* (ESOL)	−4.7	Brenk	0 alert
Solubility	8.91 × 10^−3^ mg/mL; 1.99 × 10^−5^ mol/L	Leadlikeness	No; 1 violation: MW > 350
Class	Moderately soluble	Synthetic accessibility	5.04
Log *S* (Ali)	−4.25	Toxicity and Mutagenicity	
Solubility	2.53 × 10^−2^ mg/mL; 5.64 × 10^−5^ mol/L	Carcino Mouse	Negative
Class	Moderately soluble	Carcino Rat	Negative
Log *S* (SILICOS-IT)	−6.44	Daphnia	0.08
Solubility	1.64 × 10^−4^ mg/mL; 3.66 × 10^−7^ mol/L	hERG Inhibition	Medium risk
Class	Poorly soluble	Ames test	Mutagen

**Table 2:** Binding free energy results for control and top ranked compounds at different binding sites.

Method	Energy Component	Control	Binding Site 1	Binding Site 2	Binding Site 3	Binding Site 4
MMGBSA	van der Waals Energy	−66.6851	−65.4398	−49.3253	−58.7992	−58.3329
	Electrostatic Energy	−53.9084	−12.9283	−21.8973	−14.2816	−8.3200
	Polar Solvation Energy	69.4213	32.2840	39.1614	33.5678	24.6442
	Non-polar Solvation Energy	−6.5520	−5.6700	−4.1186	−5.3352	−5.5330
	Gas Phase Energy	−120.5934	−78.3681	−71.2226	−73.0808	−66.6529
	Solvation Energy	62.8693	26.6139	35.0428	28.2325	19.1112
	Total Binding Energy	−57.7241	−51.7542	−36.1798	−44.8483	−47.5417
MMPBSA	van der Waals Energy	−66.6851	−65.4398	−49.3253	−58.7992	−58.3329
	Electrostatic Energy	−53.9084	−12.9283	−21.8973	−14.2816	−8.3200
	Polar Solvation Energy	83.5288	36.4641	42.6764	42.5941	31.4819
	Non-polar Solvation Energy	−4.4635	−3.8817	−3.6419	−3.6784	−3.9953
	Gas Phase Energy	−120.5934	−78.3681	−71.2226	−73.0808	−66.6529
	Solvation Energy	79.0654	32.5824	39.0346	38.9157	27.4866
	Total Binding Energy	−41.5280	−45.7857	−32.1881	−34.1651	−39.1663

## Data Availability

Not applicable

## References

[1] Zhu N., Zhang D., Wang W., Li X., Yang B., Song J., Zhao X., Huang B., Shi W., Lu R. (2020). A novel coronavirus from patients with pneumonia in china, 2019. N. Engl. J. Med..

[2] Wang L., Wang Y., Ye D., Liu Q. (2020). Review of the 2019 novel coronavirus (sars-cov-2) based on current evidence. Int. J. Antimicrob. Agents.

[3] Dar H.A., Waheed Y., Najmi M.H., Ismail S., Hetta H.F., Ali A., Muhammad K. (2020). Multiepitope subunit vaccine design against covid-19 based on the spike protein of sars-cov-2: An in silico analysis. J. Immunol. Res..

[4] Huang C., Wang Y., Li X., Ren L., Zhao J., Hu Y., Zhang L., Fan G., Xu J., Gu X. (2020). Clinical features of patients infected with 2019 novel coronavirus in wuhan, china. Lancet.

[5] Guan W.J., Ni Z.Y., Hu Y., Liang W.H., Ou C.Q., He J.X., Liu L., Shan H., Lei C.L., Hui D.S.C. (2020). Clinical characteristics of coronavirus disease 2019 in china. N. Engl. J. Med..

[6] Zaim S., Chong J.H., Sankaranarayanan V., Harky A. (2020). Covid-19 and multiorgan response. Curr. Probl. Cardiol..

[7] Dhama K., Sharun K., Tiwari R., Dadar M., Malik Y.S., Singh K.P., Chaicumpa W. (2020). Covid-19, an emerging coronavirus infection: Advances and prospects in designing and developing vaccines, immunotherapeutics, and therapeutics. Hum. Vaccin. Immunother..

[8] Mahmood Z., Alrefai H., Hetta H.F., H A.K., Munawar N., Abdul Rahman S., Elshaer S., Batiha G.E., Muhammad K. (2020). Investigating virological, immunological, and pathological avenues to identify potential targets for developing covid-19 treatment and prevention strategies. Vaccines (Basel).

[9] Elshabrawy H.A. (2020). Sars-cov-2: An update on potential antivirals in light of sars-cov antiviral drug discoveries. Vaccines (Basel).

[10] Wu C., Liu Y., Yang Y., Zhang P., Zhong W., Wang Y., Wang Q., Xu Y., Li M., Li X. (2020). Analysis of therapeutic targets for sars-cov-2 and discovery of potential drugs by computational methods. Acta Pharm Sin. B.

[11] Amanat F., Krammer F. (2020). Sars-cov-2 vaccines: Status report. Immunity.

[12] Chen Y., Liu Q., Guo D. (2020). Emerging coronaviruses: Genome structure, replication, and pathogenesis. J. Med. Virol..

[13] Ye Z.W., Yuan S., Yuen K.S., Fung S.Y., Chan C.P., Jin D.Y. (2020). Zoonotic origins of human coronaviruses. Int. J. Biol. Sci..

[14] Hui D.S., E I.A., Madani T.A., Ntoumi F., Kock R., Dar O., Ippolito G., McHugh T.D., Memish Z.A., Drosten C. (2020). The continuing 2019-ncov epidemic threat of novel coronaviruses to global health - the latest 2019 novel coronavirus outbreak in wuhan, china. Int. J. Infect. Dis..

[15] Phan T. (2020). Genetic diversity and evolution of sars-cov-2. Infect. Genet. Evol..

[16] Paules C.I., Marston H.D., Fauci A.S. (2020). Coronavirus infections-more than just the common cold. JAMA.

[17] Abd Ellah N.H., Gad S.F., Muhammad K., G E.B., Hetta H.F. (2020). Nanomedicine as a promising approach for diagnosis, treatment and prophylaxis against covid-19. Nanomedicine (Lond).

[18] Liu L., Wang P., Nair M.S., Yu J., Rapp M., Wang Q., Luo Y., Chan J.F., Sahi V., Figueroa A. (2020). Potent neutralizing monoclonal antibodies directed to multiple epitopes on the sars-cov-2 spike. bioRxiv.

[19] White M.A., Lin W., Cheng X. (2020). Discovery of covid-19 inhibitors targeting the sars-cov-2 nsp13 helicase. J. Phys. Chem. Lett..

[20] Habtemariam S., Nabavi S.F., Banach M., Berindan-Neagoe I., Sarkar K., Sil P.C., Nabavi S.M. (2020). Should we try sars-cov-2 helicase inhibitors for covid-19 therapy?. Arch. Med. Res..

[21] Byrd A.K., Raney K.D. (2004). Protein displacement by an assembly of helicase molecules aligned along single-stranded DNA. Nat. Struct Mol. Biol.

[22] Delagoutte E., von Hippel P.H. (2002). Helicase mechanisms and the coupling of helicases within macromolecular machines. Part i: Structures and properties of isolated helicases. Q. Rev. Biophys.

[23] Kleymann G., Fischer R., Betz U.A., Hendrix M., Bender W., Schneider U., Handke G., Eckenberg P., Hewlett G., Pevzner V. (2002). New helicase-primase inhibitors as drug candidates for the treatment of herpes simplex disease. Nat. Med..

[24] Mirza M.U., Froeyen M. (2020). Structural elucidation of sars-cov-2 vital proteins: Computational methods reveal potential drug candidates against main protease, nsp12 polymerase and nsp13 helicase. J. Pharm Anal..

[25] Jia Z., Yan L., Ren Z., Wu L., Wang J., Guo J., Zheng L., Ming Z., Zhang L., Lou Z. (2019). Delicate structural coordination of the severe acute respiratory syndrome coronavirus nsp13 upon atp hydrolysis. Nucleic Acids Res..

[26] Shu T., Huang M., Wu D., Ren Y., Zhang X., Han Y., Mu J., Wang R., Qiu Y., Zhang D.Y. (2020). Sars-coronavirus-2 nsp13 possesses ntpase and rna helicase activities that can be inhibited by bismuth salts. Virol. Sin..

[27] Briguglio I., Piras S., Corona P., Carta A. (2011). Inhibition of rna helicases of ssrna(+) virus belonging to flaviviridae, coronaviridae and picornaviridae families. Int. J. Med. Chem..

[28] Yu M.S., Lee J., Lee J.M., Kim Y., Chin Y.W., Jee J.G., Keum Y.S., Jeong Y.J. (2012). Identification of myricetin and scutellarein as novel chemical inhibitors of the sars coronavirus helicase, nsp13. Bioorg. Med. Chem. Lett..

[29] Tanner J.A., Zheng B.J., Zhou J., Watt R.M., Jiang J.Q., Wong K.L., Lin Y.P., Lu L.Y., He M.L., Kung H.F. (2005). The adamantane-derived bananins are potent inhibitors of the helicase activities and replication of sars coronavirus. Chem. Biol..

[30] Lee J.M., Cho J.B., Ahn H.C., Jung W., Jeong Y.J. (2017). A novel chemical compound for inhibition of sars coronavirus helicase. J. Microbiol. Biotechnol..

[31] Cho J.B., Lee J.M., Ahn H.C., Jeong Y.J. (2015). Identification of a novel small molecule inhibitor against sars coronavirus helicase. J. Microbiol. Biotechnol..

[32] Adedeji A.O., Singh K., Kassim A., Coleman C.M., Elliott R., Weiss S.R., Frieman M.B., Sarafianos S.G. (2014). Evaluation of ssya10-001 as a replication inhibitor of severe acute respiratory syndrome, mouse hepatitis, and middle east respiratory syndrome coronaviruses. Antimicrob. Agents Chemother..

[33] Omolo C.A., Soni N., Fasiku V.O., Mackraj I., Govender T. (2020). Update on therapeutic approaches and emerging therapies for sars-cov-2 virus. Eur. J. Pharmacol..

[34] Yu W., MacKerell A.D. (2017). Computer-aided drug design methods. Methods Mol. Biol..

[35] Nascimento Junior J.A.C., Santos A.M., Quintans-Junior L.J., Walker C.I.B., Borges L.P., Serafini M.R. (2020). Sars, mers and sars-cov-2 (covid-19) treatment: A patent review. Expert Opin. Ther. Pat..

[36] Mumtaz A., Ashfaq U.A., Ul Qamar M.T., Anwar F., Gulzar F., Ali M.A., Saari N., Pervez M.T. (2017). Mpd3: A useful medicinal plants database for drug designing. Nat. Prod. Res..

[37] Pettersen E.F., Goddard T.D., Huang C.C., Couch G.S., Greenblatt D.M., Meng E.C., Ferrin T.E. (2004). Ucsf chimera--a visualization system for exploratory research and analysis. J. Comput. Chem..

[38] Dallakyan S., Olson A.J. (2015). Small-molecule library screening by docking with pyrx. Methods Mol. Biol..

[39] Cagno V., Magliocco G., Tapparel C., Daali Y. (2020). The tyrosine kinase inhibitor nilotinib inhibits sars-cov-2 in vitro. Basic Clin. Pharmacol. Toxicol..

[40] Goodsell D.S., Morris G.M., Olson A.J. (1996). Automated docking of flexible ligands: Applications of autodock. J. Mol. Recognit..

[41] Maiorov V.N., Crippen G.M. (1994). Significance of root-mean-square deviation in comparing three-dimensional structures of globular proteins. J. Mol. Biol..

[42] Qasaymeh R.M., Rotondo D., Oosthuizen C.B., Lall N., Seidel V. (2019). Predictive binding affinity of plant-derived natural products towards the protein kinase g enzyme of mycobacterium tuberculosis (mtpkng). Plants (Basel).

[43] Vilar S., Cozza G., Moro S. (2008). Medicinal chemistry and the molecular operating environment (moe): Application of qsar and molecular docking to drug discovery. Curr. Top. Med. Chem..

[44] Daina A., Michielin O., Zoete V. (2017). Swissadme: A free web tool to evaluate pharmacokinetics, drug-likeness and medicinal chemistry friendliness of small molecules. Sci. Rep..

[45] Li X.Z., Zhang S.N., Yang X.Y. (2017). Combination of cheminformatics and bioinformatics to explore the chemical basis of the rhizomes and aerial parts of dioscorea nipponica makino. J. Pharm. Pharmacol..

[46] Karplus M., McCammon J.A. (2002). Molecular dynamics simulations of biomolecules. Nat. Struct. Biol..

[47] Case D.A., Cheatham T.E., Darden T., Gohlke H., Luo R., Merz K.M., Onufriev A., Simmerling C., Wang B., Woods R.J. (2005). The amber biomolecular simulation programs. J. Comput. Chem..

[48] Wang J., Wolf R.M., Caldwell J.W., Kollman P.A., Case D.A. (2004). Development and testing of a general amber force field. J. Comput. Chem..

[49] Yang L., Skjevik A.A., Han Du W.G., Noodleman L., Walker R.C., Gotz A.W. (2016). Data for molecular dynamics simulations of b-type cytochrome c oxidase with the amber force field. Data Brief..

[50] Izaguirre J.A., Sweet C.R., Pande V.S. (2010). Multiscale dynamics of macromolecules using normal mode langevin. Pac. Symp. Biocomput..

[51] Harvey M.J., De Fabritiis G. (2009). An implementation of the smooth particle mesh ewald method on gpu hardware. J. Chem. Theory Comput..

[52] Van Bergen L.A., Alonso M., Pallo A., Nilsson L., De Proft F., Messens J. (2016). Revisiting sulfur h-bonds in proteins: The example of peroxiredoxin ahpe. Sci. Rep..

[53] Roe D.R., Cheatham T.E. (2013). Ptraj and cpptraj: Software for processing and analysis of molecular dynamics trajectory data. J. Chem. Theory Comput..

[54] Humphrey W., Dalke A., Schulten K. (1996). Vmd: Visual molecular dynamics. J. Mol. Graph..

[55] Miller B.R., McGee T.D., Swails J.M., Homeyer N., Gohlke H., Roitberg A.E. (2012). Mmpbsa.Py: An efficient program for end-state free energy calculations. J. Chem. Theory Comput..

[56] Genheden S., Ryde U. (2015). The mm/pbsa and mm/gbsa methods to estimate ligand-binding affinities. Expert Opin. Drug Discov..

[57] Li J., Abel R., Zhu K., Cao Y., Zhao S., Friesner R.A. (2011). The vsgb 2.0 model: A next generation energy model for high resolution protein structure modeling. Proteins.

[58] Veber D.F., Johnson S.R., Cheng H.Y., Smith B.R., Ward K.W., Kopple K.D. (2002). Molecular properties that influence the oral bioavailability of drug candidates. J. Med. Chem..

[59] Arnott J.A., Planey S.L. (2012). The influence of lipophilicity in drug discovery and design. Expert Opin. Drug Discov..

[60] Lipinski C.A. (2004). Lead- and drug-like compounds: The rule-of-five revolution. Drug Discov. Today Technol..

[61] Egan W.J., Merz K.M., Baldwin J.J. (2000). Prediction of drug absorption using multivariate statistics. J. Med. Chem..

[62] Muegge I., Heald S.L., Brittelli D. (2001). Simple selection criteria for drug-like chemical matter. J. Med. Chem..

[63] Whitty A. (2011). Growing pains in academic drug discovery. Future Med. Chem..

[64] Pace C.N., Fu H., Lee Fryar K., Landua J., Trevino S.R., Schell D., Thurlkill R.L., Imura S., Scholtz J.M., Gajiwala K. (2014). Contribution of hydrogen bonds to protein stability. Protein Sci..

[65] Gurung A.B. (2020). In silico structure modelling of sars-cov-2 nsp13 helicase and nsp14 and repurposing of fda approved antiviral drugs as dual inhibitors. Gene Rep..

